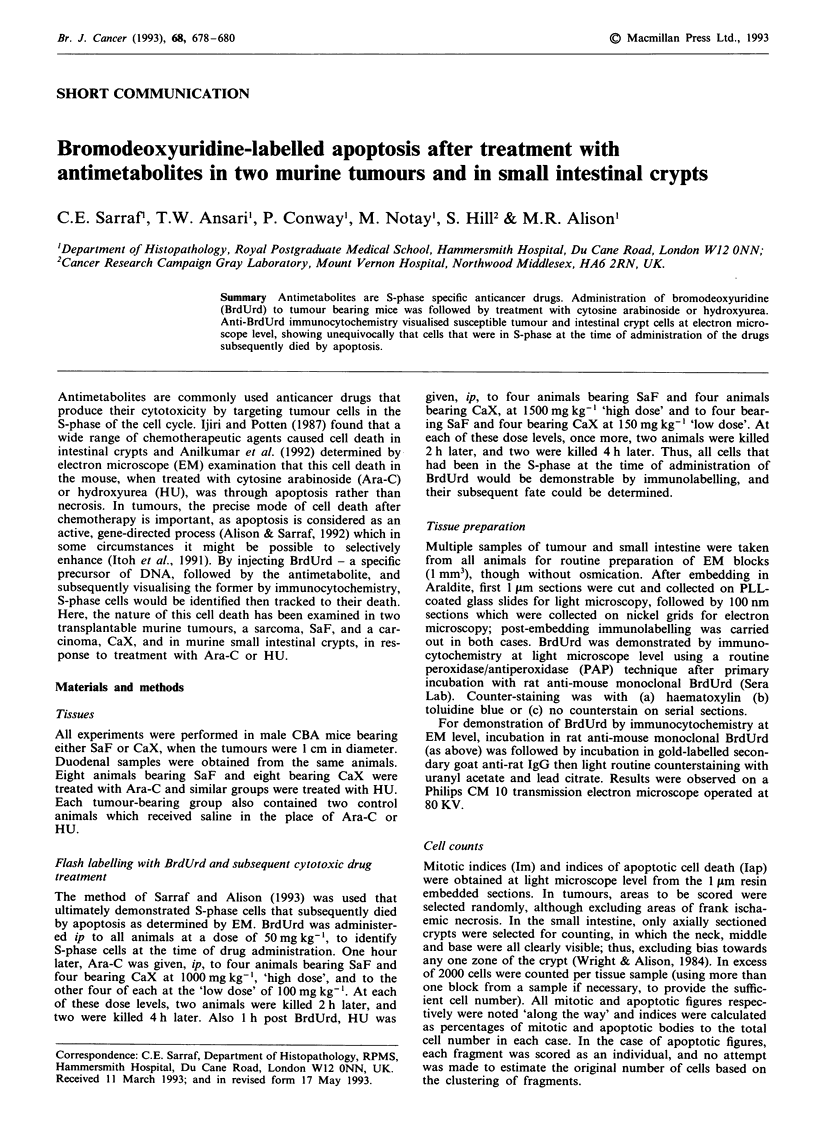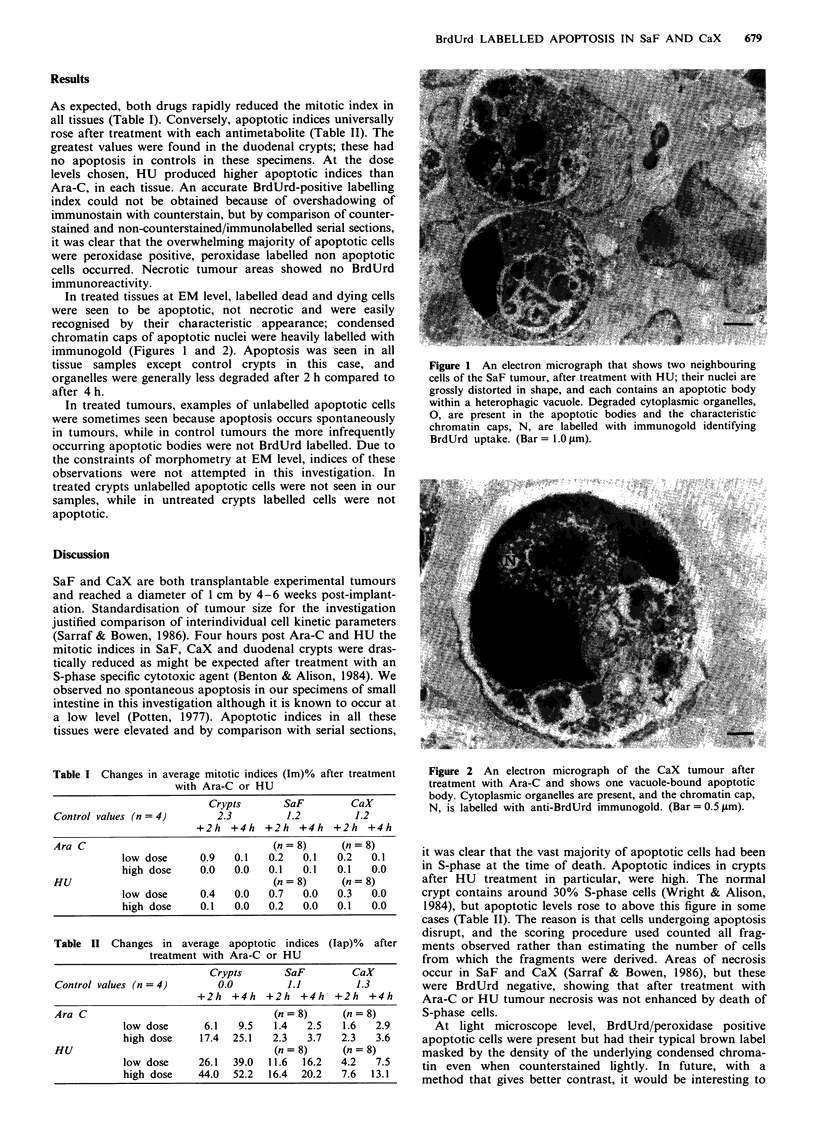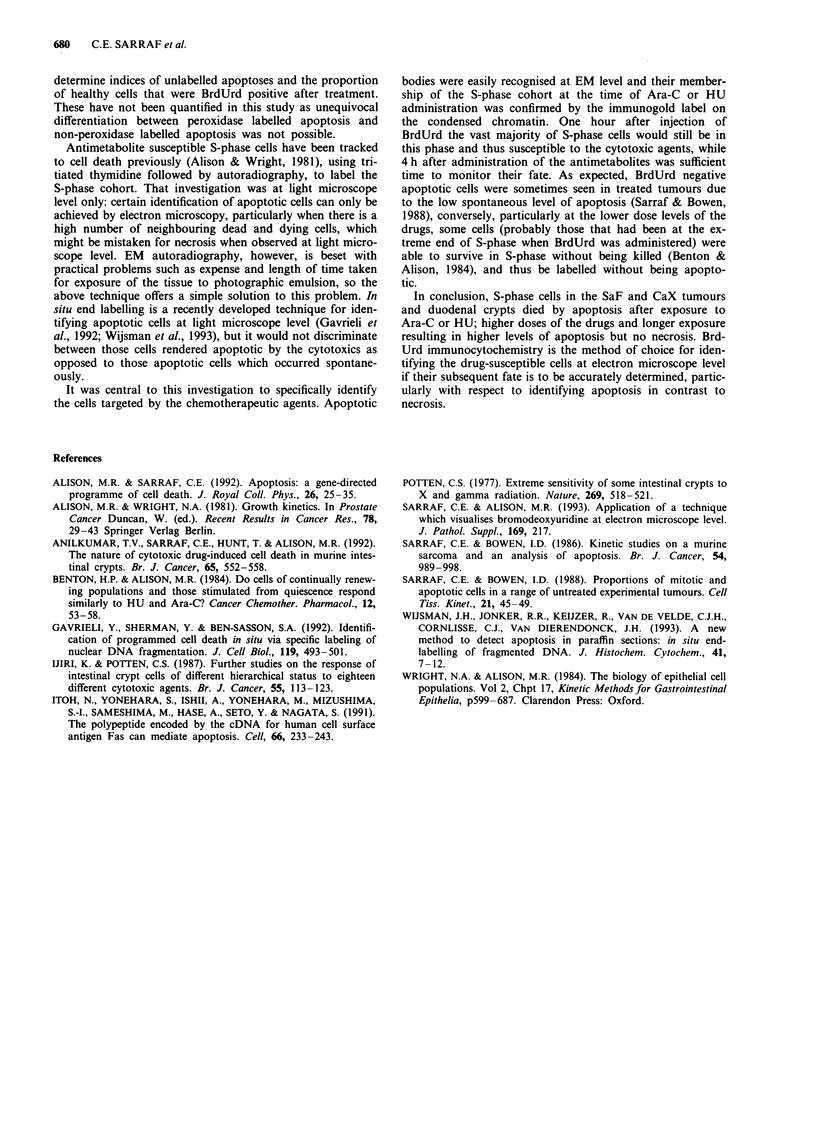# Bromodeoxyuridine-labelled apoptosis after treatment with antimetabolites in two murine tumours and in small intestinal crypts.

**DOI:** 10.1038/bjc.1993.408

**Published:** 1993-10

**Authors:** C. E. Sarraf, T. W. Ansari, P. Conway, M. Notay, S. Hill, M. R. Alison

**Affiliations:** Department of Histopathology, Royal Postgraduate Medical School, Hammersmith Hospital, London, UK.

## Abstract

**Images:**


Br. J. Cancer (1993), 68, 678–680 © Macmillan Press Ltd., 1993

SHORT COMMUNICATION

### Bromodeoxyuridine-labelled apoptosis after treatment with antimetabolites in two murine tumours and in small intestinal crypts

C.E. Sarraf[1], T.W. Ansari[1], P. Conway[1], M. Notay[1], S. Hill[2] & M.R. Alison[1]

[1]*Department of Histopathology, Royal Postgraduate Medical School, Hammersmith Hospital, Du Cane Road, London W12 0NN;* [2]*Cancer Research Campaign Gray Laboratory, Mount Vernon Hospital, Northwood Middlesex, HA6 2RN, UK.*

**Summary** Antimetabolites are S-phase specific anticancer drugs. Administration of bromodeoxyuridine (BrdUrd) to tumour bearing mice was followed by treatment with cytosine arabinoside or hydroxyurea. Anti-BrdUrd immunocytochemistry visualised susceptible tumour and intestinal crypt cells at electron microscope level, showing unequivocally that cells that were in S-phase at the time of administration of the drugs subsequently died by apoptosis.

Antimetabolites are commonly used anticancer drugs that produce their cytotoxicity by targeting tumour cells in the S-phase of the cell cycle. Ijiri and Potten (1987) found that a wide range of chemotherapeutic agents caused cell death in intestinal crypts and Anilkumar *et al.* (1992) determined by electron microscope (EM) examination that this cell death in the mouse, when treated with cytosine arabinoside (Ara-C) or hydroxyurea (HU), was through apoptosis rather than necrosis. In tumours, the precise mode of cell death after chemotherapy is important, as apoptosis is considered as an active, gene-directed process (Alison & Sarraf, 1992) which in some circumstances it might be possible to selectively enhance (Itoh *et al.*, 1991). By injecting BrdUrd – a specific precursor of DNA, followed by the antimetabolite, and subsequently visualising the former by immunocytochemistry, S-phase cells would be identified then tracked to their death. Here, the nature of this cell death has been examined in two transplantable murine tumours, a sarcoma, SaF, and a carcinoma, CaX, and in murine small intestinal crypts, in response to treatment with Ara-C or HU.

### Materials and methods

### *Tissues*

All experiments were performed in male CBA mice bearing either SaF or CaX, when the tumours were 1 cm in diameter. Duodenal samples were obtained from the same animals. Eight animals bearing SaF and eight bearing CaX were treated with Ara-C and similar groups were treated with HU. Each tumour-bearing group also contained two control animals which received saline in the place of Ara-C or HU.

### *Flash labelling with BrdUrd and subsequent cytotoxic drug treatment*

The method of Sarraf and Alison (1993) was used that ultimately demonstrated S-phase cells that subsequently died by apoptosis as determined by EM. BrdUrd was administered *ip* to all animals at a dose of 50 mg $kg^{-1}$, to identify S-phase cells at the time of drug administration. One hour later, Ara-C was given, *ip*, to four animals bearing SaF and four bearing CaX at 1000 mg $kg^{-1}$, 'high dose', and to the other four of each at the 'low dose' of 100 mg $kg^{-1}$. At each of these dose levels, two animals were killed 2 h later, and two were killed 4 h later. Also 1 h post BrdUrd, HU was given, *ip*, to four animals bearing SaF and four animals bearing CaX, at 1500 mg $kg^{-1}$ 'high dose' and to four bearing SaF and four bearing CaX at 150 mg $kg^{-1}$ 'low dose'. At each of these dose levels, once more, two animals were killed 2 h later, and two were killed 4 h later. Thus, all cells that had been in the S-phase at the time of administration of BrdUrd would be demonstrable by immunolabelling, and their subsequent fate could be determined.

### *Tissue preparation*

Multiple samples of tumour and small intestine were taken from all animals for routine preparation of EM blocks (1 $mm^3$), though without osmication. After embedding in Araldite, first 1 μm sections were cut and collected on PLL-coated glass slides for light microscopy, followed by 100 nm sections which were collected on nickel grids for electron microscopy; post-embedding immunolabelling was carried out in both cases. BrdUrd was demonstrated by immunocytochemistry at light microscope level using a routine peroxidase/antiperoxidase (PAP) technique after primary incubation with rat anti-mouse monoclonal BrdUrd (Sera Lab). Counter-staining was with (a) haematoxylin (b) toluidine blue or (c) no counterstain on serial sections.

For demonstration of BrdUrd by immunocytochemistry at EM level, incubation in rat anti-mouse monoclonal BrdUrd (as above) was followed by incubation in gold-labelled secondary goat anti-rat IgG then light routine counterstaining with uranyl acetate and lead citrate. Results were observed on a Philips CM 10 transmission electron microscope operated at 80 KV.

### *Cell counts*

Mitotic indices (Im) and indices of apoptotic cell death (Iap) were obtained at light microscope level from the 1 μm resin embedded sections. In tumours, areas to be scored were selected randomly, although excluding areas of frank ischaemic necrosis. In the small intestine, only axially sectioned crypts were selected for counting, in which the neck, middle and base were all clearly visible; thus, excluding bias towards any one zone of the crypt (Wright & Alison, 1984). In excess of 2000 cells were counted per tissue sample (using more than one block from a sample if necessary, to provide the sufficient cell number). All mitotic and apoptotic figures respectively were noted 'along the way' and indices were calculated as percentages of mitotic and apoptotic bodies to the total cell number in each case. In the case of apoptotic figures, each fragment was scored as an individual, and no attempt was made to estimate the original number of cells based on the clustering of fragments.

Correspondence: C.E. Sarraf, Department of Histopathology, RPMS, Hammersmith Hospital, Du Cane Road, London W12 0NN, UK.
Received 11 March 1993; and in revised form 17 May 1993.

### Results

As expected, both drugs rapidly reduced the mitotic index in all tissues (Table I). Conversely, apoptotic indices universally rose after treatment with each antimetabolite (Table II). The greatest values were found in the duodenal crypts; these had no apoptosis in controls in these specimens. At the dose levels chosen, HU produced higher apoptotic indices than Ara-C, in each tissue. An accurate BrdUrd-positive labelling index could not be obtained because of overshadowing of immunostain with counterstain, but by comparison of counter-stained and non-counterstained/immunolabelled serial sections, it was clear that the overwhelming majority of apoptotic cells were peroxidase positive, peroxidase labelled non apoptotic cells occurred. Necrotic tumour areas showed no BrdUrd immunoreactivity.

In treated tissues at EM level, labelled dead and dying cells were seen to be apoptotic, not necrotic and were easily recognised by their characteristic appearance; condensed chromatin caps of apoptotic nuclei were heavily labelled with immunogold (Figures 1 and 2). Apoptosis was seen in all tissue samples except control crypts in this case, and organelles were generally less degraded after 2 h compared to after 4 h.

In treated tumours, examples of unlabelled apoptotic cells were sometimes seen because apoptosis occurs spontaneously in tumours, while in control tumours the more infrequently occurring apoptotic bodies were not BrdUrd labelled. Due to the constraints of morphometry at EM level, indices of these observations were not attempted in this investigation. In treated crypts unlabelled apoptotic cells were not seen in our samples, while in untreated crypts labelled cells were not apoptotic.

### Discussion

SaF and CaX are both transplantable experimental tumours and reached a diameter of 1 cm by 4–6 weeks post-implantation. Standardisation of tumour size for the investigation justified comparison of interindividual cell kinetic parameters (Sarraf & Bowen, 1986). Four hours post Ara-C and HU the mitotic indices in SaF, CaX and duodenal crypts were drastically reduced as might be expected after treatment with an S-phase specific cytotoxic agent (Benton & Alison, 1984). We observed no spontaneous apoptosis in our specimens of small intestine in this investigation although it is known to occur at a low level (Potten, 1977). Apoptotic indices in all these tissues were elevated and by comparison with serial sections,

**Table I** Changes in average mitotic indices (Im)% after treatment with Ara-C or HU

| | | *Crypts* | | *SaF* | | *CaX* | |
|---|---|---|---|---|---|---|---|
| *Control values (n = 4)* | | *2.3* | | *1.2* | | *1.2* | |
| | | *+2 h* | *+4 h* | *+2 h* | *+4 h* | *+2 h* | *+4 h* |
| *Ara C* | | | | (*n* = 8) | | (*n* = 8) | |
| | low dose | 0.9 | 0.1 | 0.2 | 0.1 | 0.2 | 0.1 |
| | high dose | 0.0 | 0.0 | 0.1 | 0.1 | 0.1 | 0.0 |
| *HU* | | | | (*n* = 8) | | (*n* = 8) | |
| | low dose | 0.4 | 0.0 | 0.7 | 0.0 | 0.3 | 0.0 |
| | high dose | 0.1 | 0.0 | 0.2 | 0.0 | 0.1 | 0.0 |

**Table II** Changes in average apoptotic indices (Iap)% after treatment with Ara-C or HU

| | | *Crypts* | | *SaF* | | *CaX* | |
|---|---|---|---|---|---|---|---|
| *Control values (n = 4)* | | *0.0* | | *1.1* | | *1.3* | |
| | | *+2 h* | *+4 h* | *+2 h* | *+4 h* | *+2 h* | *+4 h* |
| *Ara C* | | | | (*n* = 8) | | (*n* = 8) | |
| | low dose | 6.1 | 9.5 | 1.4 | 2.5 | 1.6 | 2.9 |
| | high dose | 17.4 | 25.1 | 2.3 | 3.7 | 2.3 | 3.6 |
| *HU* | | | | (*n* = 8) | | (*n* = 8) | |
| | low dose | 26.1 | 39.0 | 11.6 | 16.2 | 4.2 | 7.5 |
| | high dose | 44.0 | 52.2 | 16.4 | 20.2 | 7.6 | 13.1 |

**Figure 1** An electron micrograph that shows two neighbouring cells of the SaF tumour, after treatment with HU; their nuclei are grossly distorted in shape, and each contains an apoptotic body within a heterophagic vacuole. Degraded cytoplasmic organelles, O, are present in the apoptotic bodies and the characteristic chromatin caps, N, are labelled with immunogold identifying BrdUrd uptake. (Bar = 1.0 μm).

**Figure 2** An electron micrograph of the CaX tumour after treatment with Ara-C and shows one vacuole-bound apoptotic body. Cytoplasmic organelles are present, and the chromatin cap, N, is labelled with anti-BrdUrd immunogold. (Bar = 0.5 μm).

it was clear that the vast majority of apoptotic cells had been in S-phase at the time of death. Apoptotic indices in crypts after HU treatment in particular, were high. The normal crypt contains around 30% S-phase cells (Wright & Alison, 1984), but apoptotic levels rose to above this figure in some cases (Table II). The reason is that cells undergoing apoptosis disrupt, and the scoring procedure used counted all fragments observed rather than estimating the number of cells from which the fragments were derived. Areas of necrosis occur in SaF and CaX (Sarraf & Bowen, 1986), but these were BrdUrd negative, showing that after treatment with Ara-C or HU tumour necrosis was not enhanced by death of S-phase cells.

At light microscope level, BrdUrd/peroxidase positive apoptotic cells were present but had their typical brown label masked by the density of the underlying condensed chromatin even when counterstained lightly. In future, with a method that gives better contrast, it would be interesting to determine indices of unlabelled apoptoses and the proportion of healthy cells that were BrdUrd positive after treatment. These have not been quantified in this study as unequivocal differentiation between peroxidase labelled apoptosis and non-peroxidase labelled apoptosis was not possible.

Antimetabolite susceptible S-phase cells have been tracked to cell death previously (Alison & Wright, 1981), using tritiated thymidine followed by autoradiography, to label the S-phase cohort. That investigation was at light microscope level only: certain identification of apoptotic cells can only be achieved by electron microscopy, particularly when there is a high number of neighbouring dead and dying cells, which might be mistaken for necrosis when observed at light microscope level. EM autoradiography, however, is beset with practical problems such as expense and length of time taken for exposure of the tissue to photographic emulsion, so the above technique offers a simple solution to this problem. *In situ* end labelling is a recently developed technique for identifying apoptotic cells at light microscope level (Gavrieli *et al.*, 1992; Wijsman *et al.*, 1993), but it would not discriminate between those cells rendered apoptotic by the cytotoxics as opposed to those apoptotic cells which occurred spontaneously.

It was central to this investigation to specifically identify the cells targeted by the chemotherapeutic agents. Apoptotic bodies were easily recognised at EM level and their membership of the S-phase cohort at the time of Ara-C or HU administration was confirmed by the immunogold label on the condensed chromatin. One hour after injection of BrdUrd the vast majority of S-phase cells would still be in this phase and thus susceptible to the cytotoxic agents, while 4 h after administration of the antimetabolites was sufficient time to monitor their fate. As expected, BrdUrd negative apoptotic cells were sometimes seen in treated tumours due to the low spontaneous level of apoptosis (Sarraf & Bowen, 1988), conversely, particularly at the lower dose levels of the drugs, some cells (probably those that had been at the extreme end of S-phase when BrdUrd was administered) were able to survive in S-phase without being killed (Benton & Alison, 1984), and thus be labelled without being apoptotic.

In conclusion, S-phase cells in the SaF and CaX tumours and duodenal crypts died by apoptosis after exposure to Ara-C or HU; higher doses of the drugs and longer exposure resulting in higher levels of apoptosis but no necrosis. BrdUrd immunocytochemistry is the method of choice for identifying the drug-susceptible cells at electron microscope level if their subsequent fate is to be accurately determined, particularly with respect to identifying apoptosis in contrast to necrosis.

### References

ALISON, M.R. & SARRAF, C.E. (1992). Apoptosis: a gene-directed programme of cell death. *J. Royal Coll. Phys.*, **26**, 25–35.

ALISON, M.R. & WRIGHT, N.A. (1981). Growth kinetics. In *Prostate Cancer* Duncan, W. (ed.). *Recent Results in Cancer Res.*, **78**, 29–43 Springer Verlag Berlin.

ANILKUMAR, T.V., SARRAF, C.E., HUNT, T. & ALISON, M.R. (1992). The nature of cytotoxic drug-induced cell death in murine intestinal crypts. *Br. J. Cancer*, **65**, 552–558.

BENTON, H.P. & ALISON, M.R. (1984). Do cells of continually renewing populations and those stimulated from quiescence respond similarly to HU and Ara-C? *Cancer Chemother. Pharmacol.*, **12**, 53–58.

GAVRIELI, Y., SHERMAN, Y. & BEN-SASSON, S.A. (1992). Identification of programmed cell death *in situ* via specific labeling of nuclear DNA fragmentation. *J. Cell Biol.*, **119**, 493–501.

IJIRI, K. & POTTEN, C.S. (1987). Further studies on the response of intestinal crypt cells of different hierarchical status to eighteen different cytotoxic agents. *Br. J. Cancer*, **55**, 113–123.

ITOH, N., YONEHARA, S., ISHII, A., YONEHARA, M., MIZUSHIMA, S.-I., SAMESHIMA, M., HASE, A., SETO, Y. & NAGATA, S. (1991). The polypeptide encoded by the cDNA for human cell surface antigen Fas can mediate apoptosis. *Cell*, **66**, 233–243.

POTTEN, C.S. (1977). Extreme sensitivity of some intestinal crypts to X and gamma radiation. *Nature*, **269**, 518–521.

SARRAF, C.E. & ALISON, M.R. (1993). Application of a technique which visualises bromodeoxyuridine at electron microscope level. *J. Pathol. Suppl.*, **169**, 217.

SARRAF, C.E. & BOWEN, I.D. (1986). Kinetic studies on a murine sarcoma and an analysis of apoptosis. *Br. J. Cancer*, **54**, 989–998.

SARRAF, C.E. & BOWEN, I.D. (1988). Proportions of mitotic and apoptotic cells in a range of untreated experimental tumours. *Cell Tiss. Kinet.*, **21**, 45–49.

WIJSMAN, J.H., JONKER, R.R., KEIJZER, R., VAN DE VELDE, C.J.H., CORNLISSE, C.J., VAN DIERENDONCK, J.H. (1993). A new method to detect apoptosis in paraffin sections: *in situ* end-labelling of fragmented DNA. *J. Histochem. Cytochem.*, **41**, 7–12.

WRIGHT, N.A. & ALISON, M.R. (1984). The biology of epithelial cell populations. Vol 2, Chpt 17, *Kinetic Methods for Gastrointestinal Epithelia*, p599–687. Clarendon Press: Oxford.